# Associations between Skeletal Growth in Childhood and Cognitive Function in Mid-Life in a 53-Year Prospective Birth Cohort Study

**DOI:** 10.1371/journal.pone.0124163

**Published:** 2015-04-15

**Authors:** Robert Stewart, Rebecca Hardy, Marcus Richards

**Affiliations:** 1 King’s College London (Institute of Psychiatry), London, United Kingdom; 2 MRC Unit for Lifelong Health and Ageing, and MRC National Survey of Health and Development, London, United Kingdom; Texas Tech University Health Science Centers, UNITED STATES

## Abstract

**Background:**

Several studies have found that shorter stature (height and limb length) in late life is associated with dementia and cognitive impairment. The extent to which childhood environment and early life cognitive function accounts for these associations is not clear.

**Methods:**

We investigated associations of adult trunk height and leg length with cognitive function in middle age, analysing data from the MRC National Survey of Health and Development: a cohort followed from birth to age 53, 1677 of whom had data on all covariates. The four cognitive tests measured verbal ability, word list memory, verbal fluency and speed/concentration. Early life environmental measures included parental education, poverty, parental divorce, physical health, cognitive ability at age 15, own education and own adult social class.

**Results:**

After adjusting for gender, shorter trunk length was associated with lower cognitive function on all four tests and shorter leg length with lower verbal intelligence and word list memory. These associations were only partially attenuated following adjustment for childhood adversity/health but were substantially accounted for by cognitive ability at age 15.

**Conclusions:**

Shorter stature was associated with lower cognitive function at age 53, the majority of this association being accounted for by cognitive function at age 15. Reduced cognitive reserve may well account for later associations between anthropometric measures and dementia.

## Introduction

Research into the aetiology of late-life cognitive impairment has highlighted the importance of risk factors across the life-course. These include aspects of the developmental environment, such as schooling,[1,2] area of residence,[3,4] parental occupation and household size in childhood.[5] These associations may be accounted for by higher education and cognitive development in early life masking evidence of cognitive decline in later life. However there is some evidence that early life cognition is associated with neuropathology in later life,[6] combined with direct associations between childhood cognition and cognitive decline,[7,8] suggesting that direct causal pathways may exist.

The early life environment may therefore be important in the etiology of cognitive function later in life. In this respect, skeletal growth is an important aspect of early life development and a marker of nutritional status in childhood.[9,10] Reduced birth weight and reduced growth in infancy are associated with slower cognitive development,[11,12] and predict a variety of adverse outcomes much later in life, such as coronary heart disease, type 2 diabetes and hypertension.[13–15] Shorter adult height,[16] and shorter leg length,[17,18] have been found to be associated with cognitive impairment and dementia. Associations between smaller head size and late-life cognitive impairment or dementia are well-recognised;[19,20] however associations between limb length and late-life cognition have been found to be independent of head size,[21] suggesting different causal pathways.

Associations between skeletal dimensions and later cognitive impairment have been found to be independent of education and social class.[17,18] However these studies have relied on recall to ascertain early life environment and may have failed to identify potential confounding factors—in particular, the quality of the childhood environment and level of childhood cognition prior to attainment of final skeletal dimensions (and which might track with skeletal growth). It has not been possible to investigate associations with trunk length in previous studies because of intervening osteodegenerative changes which make this, when measured in late life, an unreliable estimate of childhood growth. Taking advantage of a birth cohort study with a long follow-up period, we tested associations between achieved leg and trunk length and cognitive function in mid-life, and the extent to which this was accounted for by early life socio-economic status, childhood stressors and by early cognitive function.

## Materials and Methods

### Study participants

Participants were drawn from the on-going MRC National Survey of Health and Development (also known as the British 1946 birth cohort) that initially consisted of 5362 children.[22] At its inception, the sample comprised all births to non-manual and agricultural workers plus a random sample of one in four of manual workers selected from all single births within marriage that occurred in England, Wales and Scotland during one week in March, 1946. All cohort members were of white European ethnicity. Information about socio-demographic factors and medical, cognitive and psychological function has been obtained regularly over the years by interview and examination, most recently in 1999, when 3035 study members at the age of 53 years underwent interview and examination by trained research nurses. Those not interviewed at this age consisted of 469 deaths, 668 permanent refusals, 280 temporary refusals, 461 who had emigrated (with a further 119 also living abroad), and 330 who were untraced. From previously published data non-participants at age 53 were more likely in childhood to be from families in manual social class categories and were more likely to have experienced overcrowding, serious illness and late enuresis, but there was no difference between participants and non-participants in parental death or separation; however, the responders in mid-life have been found to show good representativeness on most indices in relation to the national population of a similar age.[23] Ethical approval for this research was obtained from the North Thames Multi-Centre Research Ethics Committee, and from relevant local research ethics committees in the survey areas. Informed consent was obtained from all participants.

### Measurements

#### Midlife leg and trunk length

Standing and sitting height were measured at age 53 years by trained research nurses using a stadiometer according to standard protocol, with shoes removed and head positioned in the Frankfort plane. Sitting height was used to represent trunk length, and leg length was calculated as standing height minus sitting height.

#### Midlife cognitive function

At age 53 years the following tests were administered by trained research nurses:

1. National Adult Reading Test (NART).[24] This is a pronunciation test involving 50 irregular words of increasing difficulty, chosen to violate conventional grapheme-phoneme correspondence rules. To pronounce any of the words correctly the respondent must therefore be able to recognise them in their written form rather than rely on intelligent guesswork. Thus it is effectively a test of knowledge acquisition, correlates with full-scale IQ,[24,25] and is a widely used test of crystallised intelligence in epidemiological research.
2. Verbal memory was assessed by a 15-item word learning task devised by the NSHD. Each word was shown for two seconds. When all 15 words had been shown, the cohort member was asked to write down as many of the words as possible. Total number of words correctly recalled over three identical trials was summed to provide an overall score for short term verbal memory (maximum score = 45).
3. Verbal fluency was assessed by the requirement to name as many animals as possible within one minute.
4. Speed and concentration were assessed by a visual search task, where participants were required to cross out the letters P and W, randomly embedded within a page of other letters, as quickly and accurately as possible within one minute. The score was the position reached at the end of this interval (maximum score = 600). Sensory deficits in the sample were low (<5%) at the age 53 examination.

#### Childhood cognition

At age 15 years participants took three cognitive tests: i) Group Ability Test AH4, a 130-item test requiring shape matching and selection, and verbal and number problems, yielding scores for Verbal Intelligence and Non-verbal Intelligence; ii) the Watts-Vernon Reading Test, a 35-item test of reading comprehension requiring the participant to select an appropriate word to complete a sentence; iii) a 47-item mathematics test, requiring the use of arithmetic, geometry, trigonometry and algebra. These tests of childhood cognition were combined into an aggregate total (by averaging z-scores) for use as a covariate in the present analyses, representing general cognitive ability at this age.

#### Educational attainment

The highest educational or training qualification achieved by 26 years was classified by the 8-point Burnham scale,[26] recoded into no qualification, below ordinary secondary qualifications (vocational), ordinary secondary qualifications (‘O’ levels and their training equivalents; implying at least 11 years school attendance), advanced secondary qualifications (‘A’ levels and their equivalents; implying at least 13 years school attendance), or higher qualifications (degree or equivalent).

#### Maternal education

Maternal education was classified into those with primary or secondary education only with no formal qualifications versus those with qualifications or any further education.

#### Occupational social class

Occupational social class of origin (when participants were aged 11 years, or if this was unknown, at four or 15 years) and current or last own occupation at age 43 years were assigned according to the Registrar General (RG) system,[27] classified as professional (I), managerial/intermediate (II), skilled non-manual (IIInm), skilled manual (IIIm), semi-skilled manual (VI), and unskilled (V), then dichotomized into manual versus non-manual.

### Early adversity variables.

1. Material home conditions at 4 years

An aggregate variable representing material home conditions at age four years was obtained by summing the following ratings (scored 0 and 1), made during the home-based interview by a health visitor:

- State of repair of house (1 = very good)
- Age of house (1 = built post 1919)
- Crowding (1 = less than 1.5 persons per room)
- Cleanliness of house (1 = very clean)
- Cleanliness of survey member (1 = very clean)
- Condition of survey member’s shoes (1 = adequate)
- Condition of survey member’s clothes (1 = adequate)

To produce a manageable indicator variable, this total score was recoded into four categories, i.e. very good (score = 7), good (score = 5–6), modest (score = 3–4), and poor (score = 0–2).

2. Parental divorce up to 15 years

Because of the relatively small numbers of survey members who had experienced parental divorce by this age, the indicator variable was dichotomised to absent vs. present.

3. Maternal management and understanding at four years

As with the material home condition variables, maternal management and understanding was rated during the home-based interview by a health visitor, and scored as among the best, average, or among the worst. Because of the small cell size for the worst category, this was combined with the average category. Thus the indicator variable was dichotomised to Good vs. Average/Poor.

4. Medical disorder by age 5 years

ICD-8 diagnoses by age 5 years were recorded. These represented the following disorders: infectious, neoplasm, endocrine, nutritional, metabolic, neurological, cognitive, sensory, circulatory, digestive, genitourinary, skin, musculoskeletal, congenital, accident, and ill-defined. These were recoded to any versus none.

### Statistical analysis

The dataset was restricted to the sub-sample with complete data on all variables under consideration. Initial analyses were carried out dividing leg and trunk length by quintiles to check visually for non-linearity. Subsequent analyses modelled these variables continuously using the standard deviation as a unit to improve clarity and comparability of coefficients—i.e. anthropometric measures were ‘z-transformed’ by subtracting the sample mean and dividing by the sample standard deviation. Eight linear regression models were used to investigate the association between each of the two (z-transformed) anthropometric measures with each of the four cognitive test scores at age 53. These associations with adult leg and trunk length were sequentially adjusted for covariates. The first models adjusted for sex alone; the second model additionally entered all early adversity measures as covariates; the third model additionally entered cognition at age 15; and the fourth model additionally entered education and social class. Secondary analyses tested associations between early life adversity and cognitive test scores at age 53 and compared the size of the regression coefficients before and after separate adjustment for adult leg and trunk length. Stata software was used.

## Results

The sample size with non-missing data for all variables in this study was 1677. Those with any missing data at age 53 years had significantly lower childhood cognitive scores, were more likely to be male, were more likely to be from relatively disadvantaged circumstances of origin (manual social class father, mother with no educational qualifications and sub-optimum parenting skills, poorer material home circumstances) and with lower educational and occupational attainment, compared to those in the analysis (p < 0.001 in all cases, data not shown). There was no difference between these two groups in the frequency of parental divorce (p = 0.13).

Unadjusted associations with cognitive function at age 53 are summarised in Table 1. Ascending fifths of trunk length were significantly associated with higher verbal ability and verbal fluency, while ascending fifths of leg length were only associated with higher verbal ability. No significant deviation from linearity in these associations was found. Apart from the association between maternal management and letter search speed, all four tests were significantly associated with higher education, higher occupational social class, higher cognitive ability at age 15, better material home conditions, better rated maternal management, higher maternal education and higher paternal social class. Verbal memory and letter search scores were higher in women, and lower verbal ability and verbal memory were associated with parental divorce.

**Table 1:** Mean midlife cognitive test scores by all covariates.

Covariates		Mean (SE) cognitive test score at age 53			
		Verbal ability (NART)	Verbal memory	Verbal fluency	Letter search
Leg length (quintiles)	1 (n = 338)	33.01 (0.53)	24.12 (0.35)	23.28 (0.36)	285.50 (4.22)
	2 (n = 330)	33.78 (0.53)	24.44 (0.35)	24.31 (0.37)	276.85 (3.91)
	3 (n = 333)	34.16 (0.48)	24.50 (0.31)	23.83 (0.39)	277.68 (3.84)
	4 (n = 334)	35.01 (0.48)	23.70 (0.36)	24.77 (0.38)	277.16 (4.12)
	5 (n = 332)	35.85 (0.52)	23.38 (0.35)	24.14 (0.36)	276.26 (4.13)
Trunk length (quintiles)	1 (n = 340)	32.87 (0.52)	24.05 (0.37)	23.37 (0.38)	281.44 (3.90)
	2 (n = 345)	33.61 (0.51)	23.94 (0.33)	23.92 (0.37)	277.84 (4.09)
	3 (n = 323)	34.71 (0.51)	24.42 (0.34)	24.03 (0.36)	279.22 (4.10)
	4 (n = 337)	34.34 (0.49)	23.37 (0.32)	23.97 (0.37)	277.70 (4.13)
	5 (n = 332)	36.18 (0.50)	24.32 (0.34)	24.98 (0.38)	277.15 (3.95)
Gender	Male (n = 829)	34.60 (0.32)	23.10 (0.21)	24.10 (0.23)	270.83 (2.52)
	Female (n = 848)	34.06 (0.32)	24.91 (0.22)	24.01 (0.24)	286.34 (2.55)
Father’s social class	Manual (n = 708)	31.63 (0.30)	22.74 (0.20)	22.99 (0.21)	271.83 (2.29)
	Non-manual (n = 969)	38.01 (0.30)	25.76 (0.23)	25.50 (0.26)	288.04 (2.96)
Mother’s education	Primary only (n = 1029)	32.16 (0.29)	23.03 (0.19)	23.28 (0.21)	273.21 (2.18)
	Qualifications (n = 648)	37.76 (0.33)	25.58 (0.24)	25.28 (0.27)	287.35 (3.10)
Parental divorce by age 15	No (n = 1583)	34.43 (0.23)	24.12 (0.16)	24.06 (0.17)	278.94 (1.86)
	Yes (n = 94)	32.52 (1.02)	22.18 (0.64)	23.96 (0.66)	274.16 (7.62)
Maternal management	Optimal (n = 886)	36.30 (0.30)	25.15 (0.21)	24.73 (0.23)	281.96 (2.52)
	Sub-optimal (n = 791)	32.11 (0.33)	22.75 (0.21)	23.29 (0.24)	274.99 (2.57)
Material home conditions	Very good (n = 450)	36.51 (0.38)	25.34 (0.30)	25.16 (0.34)	288.10 (3.78)
	Good (n = 686)	35.22 (0.36)	24.38 (0.24)	24.45 (0.26)	282.58 (2.78)
	Modest (n = 431)	32.33 (0.43)	22.96 (0.28)	22.94 (0.30)	270.67 (3.24)
	Poor (n = 110)	27.65 (0.94)	20.45 (0.59)	21.35 (0.60)	247.07 (6.40)
Medical disorder by age 5	No (n = 1593)	34.37 (0.23)	24.07 (0.16)	24.16 (0.17)	278.98 (1.86)
	Yes (n = 84)	33.55 (1.20)	22.94 (0.70)	22.04 (0.67)	272.83 (7.40)
Cognitive ability at age 15 (tertiles)	1 (n = 575)	27.12 (0.36)	20.03 (0.22)	21.14 (0.25)	262.73 (2.98)
	2 (n = 549)	35.34 (0.31)	24.54 (0.24)	24.21 (0.26)	278.51 (3.01)
	3 (n = 553)	40.81 (0.26)	27.64 (0.23)	26.92 (0.30)	295.40 (3.23)
Educational attainment by age 26	Standard^1^ (n = 1058)	31.07 (0.28)	22.45 (0.18)	22.91 (0.20)	272.82 (2.20)
	Advanced^2^ (n = 619)	39.89 (0.28)	26.69 (0.23)	26.01 (0.28)	288.68 (3.09)
Social class at age 43	Manual (n = 1172)	28.52 (0.42)	20.65 (0.26)	22.10 (0.29)	264.17 (3.19)
	Non-manual (n = 505)	36.83 (0.24)	25.46 (0.17)	24.89 (0.20)	284.92 (2.16)

^1^Ordinary (‘O’) level or lower, implying 11 years or lower education.

^2^Advanced (‘A’) level or higher, implying 12 years or more education.

Adjusted associations between continuously distributed anthropometric measures and mid-life cognitive function are summarised in Tables 2 and 3. After adjustment for sex, increased leg length was associated with higher verbal ability and verbal memory. Both were reduced substantially in strength following adjustment for early life circumstances and were close to the null after further adjustment for cognition at age 15 years. Associations were found between increased trunk length and higher performance on all four tests after adjustment for sex, but these also were reduced substantially after further adjustment for early life circumstances and then again after adjustment for cognition at age 15.

**Table 2:** Linear regression analysis of associations between leg length and mid-life cognitive function.

Adjustment	Linear regression B-coefficient per SD increase in leg length (95% CI)			
	Verbal ability (NART)	Verbal memory	Verbal fluency	Letter search
Sex only	**0.14 (0.08, 0.21), p<0.001**	**0.08 (0.02, 0.14), p = 0.01**	0.04 (-0.02, 0.11), p = 0.15	0.03 (-0.04, 0.09), p = 0.40
+ early circumstances	**0.07 (0.02, 0.13), p = 0.01**	0.02 (-0.03, 0.08), p = 0.42	0.008 (-0.05, 0.07), p = 0.80	<0.001 (-0.06, 0.06), p = 0.99
+ cognition at 15 yr	0.02 (-0.02, 0.07), p = 0.28	-0.02 (-0.07, 0.03), p = 0.48	-0.02 (-0.08, 0.04), p = 0.51	-0.01 (-0.07, 0.05), p = 0.71
+ education and adult social class	0.02 (-0.03, 0.06), p = 0.44	-0.02 (-0.07, 0.03), p = 0.34	-0.02 (-0.08, 0.04), p = 0.48	-0.01 (-0.07, 0.05), p = 0.67

**Table 3:** Linear regression analysis of associations between trunk length and mid-life cognitive function.

Adjustment	Linear regression B-coefficient per SD increase in trunk length (95% CI)			
	Verbal ability (NART)	Verbal memory	Verbal fluency	Letter search
Sex only	**0.13 (0.07, 0.18), p<0.001**	**0.13 (0.07, 0.18), p<0.001**	**0.10 (0.05, 0.16), p<0.001**	**0.06 (0.01, 0.12), p = 0.03**
+ early circumstances	**0.08 (0.02, 0.13), p = 0.004**	**0.09 (0.04, 0.14), p = 0.001**	**0.07 (0.02, 0.13), p = 0.01**	0.04 (-0.02, 0.10), p = 0.16
+ cognition at 15 yr	0.01 (-0.03, 0.05), p = 0.59	0.04 (-0.01, 0.08), p = 0.12	0.04 (-0.02, 0.09), p = 0.18	0.02 (-0.03, 0.08), p = 0.38
+ education and adult social class	0.01 (-0.03, 0.05), p = 0.56	0.04 (-0.01, 0.08), p = 0.12	0.04 (-0.02, 0.09), p = 0.17	0.02 (-0.03, 0.08), p = 0.37

Gender x trunk and gender x leg length interactions were significant at the 5% level for the association between trunk length and the NART, and between leg length and letter search speed. Post hoc analyses revealed that both associations were stronger in males. For trunk length and the NART, unadjusted coefficients were 0.18 (0.10, 0.26), p<0.001 for males and 0.07 (-0.01, 0.15), p = 0.08 for females; fully adjusted these were 0.06 (0.005, 0.12), p = 0.03 for males and -0.04 (-0.09, 0.02), p = 0.17 for females. For leg length and letter search speed unadjusted coefficients were 0.12 (0.03, 0.20), p = 0.007 for males and -0.07 (-0.15, 0.02), p = 0.14 for females; fully adjusted these were 0.05 (-0.03, 0.13), p = 0.24 for males and -0.08 (-0.17, 0.006), p = 0.07 for females. No other gender x trunk or leg length interactions reached significance at the 10% level or less.

Further analyses were carried out to investigate the extent to which associations between early life circumstances (SES, parental divorce, health, material home circumstances and maternal management) and mid-life cognitive function were accounted for by leg/trunk length. The strengths of these associations were all very similar before and after separate adjustments for leg and trunk length (data not shown).

## Discussion

The importance of the early life environment in determining later risk of cognitive impairment is well recognised, although the majority of research has focussed on education as an exposure. Cognitive reserve theory remains the most accepted explanation for this, whereby level of cognitive function determined by early adulthood (although possibly modified by later experiences) provides a buffer against later deterioration so that the onset of clinically significant symptomatology is delayed. Beyond education, aspects of the early life environment influencing later cognition are poorly understood. However, growth in childhood is associated with cognitive development in this cohort[28] and elsewhere,[29] and several studies have found associations between anthropometric measures and cognitive impairment or dementia. The majority of these have investigated head circumference,[20,30–32] but associations have also been found for other measures of skeletal growth including height,[16,33–35] arm span,[17,34,36] and leg length,[17,18,21,34,36] all of which show smaller dimensions in older people with dementia or cognitive impairment compared to those without. We therefore sought to understand better the relationship between skeletal dimensions and mid-life cognitive function, taking into account both the childhood environment, insofar as measures were available on this, and cognitive function attained by early adulthood—to clarify whether the later associations reflected those apparent earlier in life, or were potentially accounted for by later experiences.

Mechanisms underlying associations between smaller skeletal size and cognitive function have yet to be established. Although the two are correlated, adjustment for head size does not appear to account in late-life for the association between shorter leg length and dementia,[21] suggesting that they may be markers of independent underlying causal processes. However, this has not been investigated in mid-life and measures of head size were not available in the cohort described here. Clearly both skeletal size and cognitive function have genetic determinants; however, *common* genetic determinants have not been reported, and environmental factors are thus more supported by evidence to date. Adult height is known to be strongly influenced by nutritional status and the wider social environment in childhood. Limb length is particularly influenced by this: for example, intergenerational increases in height in Japan were predominantly accounted for by leg length rather than trunk height.[37] Anthropometric measures have previously been investigated in relation to childhood adversity: leg growth was found to be particularly strongly associated with early life nutrition such as breast feeding and energy intake at age 4, while trunk growth was associated with later childhood stressors such as illness and parental divorce but not with nutritional status.[9] In the analyses reported here, various measures of adversity were considered as covariates, but nutritional measures were not felt to be adequate in the dataset for evaluation—a limitation of the study which should be borne in mind.

Significant associations were found between shorter trunk length and lower mid-life cognitive function, most of which remained significant after adjustment for sex and early life adversity but all of which were substantially attenuated by cognitive ability at age 15. The association itself is consistent with the findings mentioned earlier of associations between shorter height and late-life cognitive impairment, but suggests that a substantial component of this is accounted for by tracking of earlier cognitive function, in this case at age 15 years, as well as by the likely relationship between mid-life cognitive function and childhood IQ. Childhood IQ is likely to be determined before the attainment of full trunk and leg length—i.e. skeletal growth may be a *marker* of childhood cognitive development (and therefore cognition in later adulthood) rather than being an independent risk factor for later cognition. However, leg and trunk length data were not unfortunately available at age 15 in this cohort to investigate the issue further. Direct causal associations between early-life growth and neurodegenerative pathologies manifesting in later life are unlikely since, if present at all, these would have a negligible influence on cognitive function in this cohort at the age of examinations. Furthermore, a post mortem study found no associations between smaller head circumference (or lower education) and Alzheimer pathology despite an association with dementia,[32] and another prospective study found that shorter stature was associated only with cognitive impairment and not with cognitive decline.[18]

Contrary to associations found in samples in late life, associations between leg length and cognitive function were relatively weak in this cohort compared to those with trunk length. It is possible that associations with attained trunk length remain strong into late life but are obscured because of intervening osteodegenerative disorders which influence trunk (rather than leg) length in older age groups, introducing measurement error. It is also possible that associations with the different components of adult height vary between cohorts. Furthermore, if the two are influenced in different ways by different aspects of childhood environment, as has been suggested,[9] then it is possible that early life influences on cognition may vary across the life course. These questions require further research.

Study limitations should be noted. First, there was disproportionate loss to follow-up of those who were relatively disadvantaged, including those with missing data for the present analyses, and it is possible that people with combinations of low skeletal growth and lower cognitive function were under-represented, obscuring associations of interest; however, the *patterns* of associations (e.g. the role of covariate adjustments) would be unlikely to be influenced by such selection. Second, the cohort has so far only reached mid-life, and inferences regarding possible associations later in life are limited. Third, the covariates were clearly restricted to measurements which had been administered in the past in this long-running cohort (and, as mentioned, focused more on adversity than nutrition). However, a major strength was the large well-characterised sample which, uniquely in international research, had been followed since birth with prospectively collected data on childhood circumstances substantially reducing the problems of recall bias. Direct measurements had been made of anthropometric measures, and cognitive function had been assessed across several domains.
